# Cognitive Profile Discrepancies among Typical University Students and Those with Dyslexia and Mixed-Type Learning Disorder

**DOI:** 10.3390/jcm12227113

**Published:** 2023-11-15

**Authors:** Maristella Scorza, Samuel T. Gontkovsky, Marta Puddu, Angela Ciaramidaro, Cristiano Termine, Loriana Simeoni, Marcella Mauro, Erika Benassi

**Affiliations:** 1 Department of Biomedical, Metabolic and Neural Sciences, University of Modena and Reggio Emilia, 42121 Reggio Emilia, Italy; angela.ciaramidaro@unimore.it (A.C.); erika.benassi@unimore.it (E.B.); 2 Adena Health System, The Ohio State University, Chillicothe, OH 45601, USA; s.gontkovsky@adena.org; 3 Independent Researcher, 40100 Bologna, Italy; martapuddu@psypec.it; 4 Department of Medicine and Technological Innovation, University of Insubria, 21100 Varese, Italy; cristiano.termine@uninsubria.it; 5 Child and Adolescent Neuropsychiatry Unit, ASST dei Sette Laghi, 21100 Varese, Italy; loriana.simeoni@asst-settelaghi.it; 6 Humanitas Medical Care, 20089 Milan, Italy; marcella.mauro@mc.humanitas.it

**Keywords:** WAIS-IV, cognitive profile, mixed-type SLD, dyslexia, university students

## Abstract

Background: Previous studies have identified areas of cognitive weakness in children diagnosed with Specific Learning Disorder (SLD), in the areas of working memory and processing speed in particular. In adulthood, this literature is still scant, and no studies have compared the cognitive profile of university students with dyslexia (DD) with that of students with Mixed-type SLD. Method: Thus, in this study, the WAIS-IV was used to examine the cognitive functioning of three groups of university students: students with DD, with Mixed-type SLD, and typical students. Statistical analyses were performed to examine differences in WAIS-IV FSIQ, main, and additional indexes and subtests. Results: The results showed strengths in perceptual reasoning and good verbal comprehension abilities in both the DD and Mixed-type SLD group, with weaknesses in working memory and processing speed, leading to a pattern of a better General Ability Index (GAI) than Cognitive Proficiency Index (CPI) in both clinical groups. Thus, discrepancies between GAI and CPI, well documented in children with SLD, still manifest in adulthood in university students. Our findings also revealed worse cognitive performance in university students with mixed learning disorder relative to students with only a reading deficit. Conclusions: The cognitive features and distinctive subtest profiles that emerged should guide the assessment and the definitions of intervention programs, special educational needs, and strategies of compensation.

## 1. Introduction

Specific Learning Disorders (SLDs) are a group of neurodevelopmental disorders that become apparent in school-age children. The SLDs manifest as persistent difficulties in reading, spelling, writing, and/or arithmetic skills. SLDs are neither a mere consequence of poor instruction or socio-cultural deprivation nor due to intellectual disability, sensory impairments, or acquired conditions, and persist despite conventional teaching [1,2]. These disorders are lifelong conditions and may cause significant problems, such as difficulties in academic self-efficacy [3,4,5], poor cognitive profile [6,7,8,9,10], and low quality of life [11,12,13,14] or psychological well-being [15,16,17,18,19].

The evaluation of intellectual abilities with standardized intelligence tests is a necessary step during the diagnostic process of SLD, to exclude the presence of intellectual disability and to identify individual cognitive strengths and weaknesses. The Wechsler Adult Intelligence Scale—Fourth Edition (WAIS-IV) [20,21] is one of the most popular measures of Intelligence Quotient (IQ) in Europe and the U.S.A. and is often used in neuropsychological assessments [22]. The WAIS-IV assesses performance on four cognitive domains, including verbal comprehension, perceptual reasoning, working memory, and processing speed, which determine the Full-Scale Intelligent Quotient (FSIQ). The combined IQ score, thus, provides a reliable measure of the cognitive profile of the student. In addition to the FSIQ, it is also possible to calculate the General Ability Index (GAI) and Cognitive Proficiency Index (CPI). The GAI includes the Verbal Comprehension Index (VCI) and the Perceptual Reasoning Index (PRI) and serves as an alternative global estimate of intelligence for individuals whose working memory and processing speed indexes are significantly lower than VCI and PRI [23,24]. The CPI includes the subtests that form the working memory index (WMI) and the processing speed index (PSI); the CPI is proposed as both a measure of efficiency taking in and responding to information and facility in holding and manipulating information mentally, which are thought to enhance learning and problem solving [25,26].

A large number of studies have explored the usefulness of WAIS-IV in identifying specific intellectual profiles in adults with different disorders, such as attention deficit hyperactivity disorders (e.g., [27,28]), autism spectrum disorder (e.g., [29,30]), intellectual disability (e.g., [22,31,32]), neurological disorders (e.g., [33,34,35]), and mental diseases (e.g., [36,37]). The results of these studies showed different cognitive profiles with strengths and weaknesses depending on the clinical conditions.

With regard to the cognitive profile in students with SLD, it has been extensively investigated in childhood and adolescence, and much less in adulthood. Some studies found in these children an impaired performance on subtests that evaluated working memory and processing speed, whereas they had normal verbal comprehension and perceptual reasoning indexes, thus preserving GAI [38,39,40,41,42,43,44]. For some authors the GAI may be a valid alternative way of summarizing the overall intellectual functioning of children with SLD [45,46], unlike the FSIQ which appears to be affected by processing deficits [47,48,49,50]. In addition, some authors showed that the WISC-IV [51] intellectual profile differed across SLD subtypes. For example, Poletti [42] reported that reading impairment is associated with difficulties in processing speed, while mathematical deficits are associated with a more general cognitive deficit involving fluid intelligence, working memory, and processing speed. Toffalini and colleagues [44] showed a similar GAI–CPI discrepancy across all four groups of children (children with reading disorder, spelling disorder, disorder of arithmetical skills, and mixed disorder): all SLD subgroups shared similar weaknesses in working memory and processing speed, and thus in the CPI. However, the SLD subtypes also showed some differences with each other: the deficit in the CPI was only a few points below the normative data in the children with reading and spelling disorders, and around 1 SD below the normality in the children with mixed disorder; furthermore, the PRI was higher in the children with reading disorder than in the other groups, and it appeared relatively low in the children with arithmetic disorder.

The studies that have investigated the cognitive profile using the WAIS-IV in adults with SLD are much fewer than those on children, and report less clear results. Gontkovsky and Golden [52] conducted a pilot investigation of WAIS-IV subtest profiles in two groups of adults between 20 and 62 years of age characterized by learning disorders in reading and mathematics. These authors found that individuals with mathematical deficit performed significantly lower on FSIQ and on all WAIS-IV indexes, except for the VCI, relative to the normative group; the adults with reading disorder showed significantly lower scores than the normative group only on the WMI. Furthermore, the Arithmetic and Digit Span subtests were within the three lowest scores for both groups, the differentiating lower score between groups being Block Design for the group with mathematic disorder and Symbol Search for the group with reading disorder. Thus, the authors suggested that adults with SLD with impairments in mathematics and reading show distinctive subtest profiles, namely a BAD (Block Design, Arithmetic, and Digit Span) profile for the first group and a SAD (Symbol Search, Arithmetic, and Digit Span) profile for the second group. Franzen and colleagues [53] investigated the PSI of adults with dyslexia (DD) and found that they had slower processing speed on the Coding subtest but not on the Symbol Search subtest compared to the control group. To our knowledge, only one study [54] has investigated the cognitive profile using the WAIS-IV in university students with DD. D’Elia and colleagues [54] conducted an exploratory study comparing the cognitive profile of these students with that of university students with Reading Comprehension Disorder (RCD). The findings of this Italian study demonstrated that the students with DD displayed higher scores on VCI and, as a result, on GAI than students with RCD; on the other hand, the students with DD showed lower scores on WMI, PSI, and, as a result, on CPI. Some studies that investigated the cognitive profile of adults with DD based on previous versions of the WAIS (version R or III), revealed that individuals with DD had a lower Verbal IQ compared with Performance IQ; moreover, those with higher education level were found to perform better on verbal tests, thus demonstrating less discrepancy between Verbal IQ and Performance IQ [55,56,57].

Given the scarcity of studies in adults with SLD, in this study we aimed to examine the intellectual profile using the WAIS-IV in a sample of university students, comparing two groups of students with DD and with Mixed-type SLD with a control group. In line with the literature reviewed above [52,54], we hypothesized weaknesses in working memory and processing speed indexes (WMI and PSI), leading to a pattern of a better GAI than CPI in both the clinical groups. We also hypothesized worse performance in the Mixed-type SLD group [44]. Moreover, we reported strengths in perceptual reasoning (PRI) and good VCI in both clinical groups, them being university students [55,56]. Ultimately, we expected that the discrepancies between GAI and CPI, well documented in children with SLD, may still manifest in adulthood in university students.

## 2. Materials and Methods

### 2.1. Participants

Ninety-two university students between 17.9 and 27.1 years of age took part in the study. Of these, 38 were typically developing (TD) students (Mean age = 22.0, SD = 1.7; female = 63.2%); 16 were diagnosed clinically with Dyslexia (DD group) and had a mean age of 20.8 (SD = 2.7; females = 75%); 38 students had a diagnosis of DD associated to another SLD, i.e., dysorthography and/or dysgraphia and/or dyscalculia (Mixed-SLD group) and a mean age of 19.4 (SD = 1.2; females = 50%). Specifically, the Mixed-SLD group consisted of 14 students with DD, dysorthography and dyscalculia; 12 students with DD and dysorthography; 6 students with DD and dyscalculia; 2 students with DD, dysorthography, dysgraphia, and dyscalculia; 2 students with DD, dysorthography and dysgraphia; 2 students with DD, dysgraphia and dyscalculia. The three groups significantly differed in age [*F*(2,91) = 24.30, *p* < 0.001] with the following *p* values (DD group/TD group: *p* = 0.031; Mixed-SLD group/TD group: *p* < 0.001; DD group/Mixed-SLD group: *p* = 0.022). There were no significant differences among them in sex [Chi-square (1, *N* = 92) = 1.35, *p* = 0.245].

All participants were university students or fifth-grade high school students pre-enrolled at university. Students with DD and Mixed-SLD were recruited through clinical centers and the University of Modena and Reggio Emilia. All participants received a diagnosis based on the ICD-10 [2] coding system and met the criteria indicated in the National Italian Consensus Conference on SLD published by the Italian Ministry of Health [58]. The controls were randomly selected among university students of the University of Modena and Reggio Emilia. They received an interview with a psychologist before their recruitment in the study; reading, writing, and arithmetic abilities were also evaluated. None of the participants had visual or hearing impairments, neurological or psychiatric problems, and all of them were Italian native speakers. 

### 2.2. Procedure and Measure

One task was employed for the collection of data: the Wechsler Adult Intelligence Scale—Fourth Edition (WAIS-IV) [20,21] Italian edition [59]. This scale is designed to evaluate a variety of cognitive abilities for people between 16 and 90 years old. It is employed to measure the Full-Scale IQ (FSIQ) and four Index scores: Verbal Comprehension Index (VCI), Perceptual Reasoning Index (PRI), Working Memory Index (WMI), and Processing Speed Index (PSI). The WAIS-IV is composed of 10 core subtests: Vocabulary (VC), Information (IN), and Similarities (SI) which compose the VCI; Block Design (BD), Matrix Reasoning (MR), and Visual Puzzles (VP) which determine the PRI; Digit Span (DS) and Arithmetic Reasoning (AR) which compose the WMI; Coding (CD) and Symbol Search (SS) which determine the PSI. It also contains five optional subtests: Comprehension, Letter–Number Sequencing, Figure Weights, Picture Completion, and Cancellation.

The test was individually administered to the participants in a quiet room during one unique session of about an hour and a half. The whole testing was carried out by a trained psychologist. We presented the 10 core subtests to all subjects of the three groups. We then calculated the FSIQ (VCI+PRI+WMI+PSI weighted scores then converted to IQ), VCI (VC+IN+SI weighted scores then converted to Index), PRI (BD+MR+VP weighted scores then converted to Index), WMI (DS+AR weighted scores then converted to Index), and PSI (CD+SS weighted scores then converted to Index) for each group. We also calculated the scores for the two additional indexes: the General Ability Index (GAI), sum of the VCI and PRI weighted scores then converted to Index (see conversion Tables in [23]); and the Cognitive Proficiency Index (CPI), sum of the WMI and PSI weighted scores then converted to Index (see conversion Tables in [60]). The GAI serves as an alternative global estimate of intelligence for an individual whose PSI and WMI are significantly lower than VCI and PRI [23,24]. The CPI is proposed as both a measure of efficiency in taking in and responding to information and facility in holding and manipulating information mentally, which are thought to enhance learning and problem solving [25,26]. Additional information on the subtests, main factor indexes, and additional indexes are available elsewhere [20,21,23,59,60]. The study met the ethical guidelines for human subject protection, including adherence to the legal requirements of the country (Declaration of Helsinki), and it received formal approval by the local research Ethical Committee of Area Vasta Emilia Nord, Italy (protocol code 2023/0044335 on 6 April 2023). 

### 2.3. Statistical Analysis

All statistical analyses were carried out using SPSS 27.0 for Windows. With regard to FSIQ and indexes, preliminary analyses of data distribution showed that all these variables were normally distributed (Kolmogorov–Smirnov test) and demonstrated homogeneity of variance (Levene test). Therefore, parametric analyses were applied. One univariate analysis of variance (ANOVA) controlling for age was performed to compare the three groups on FSIQ. Two multivariate analyses of variance (MANOVA) controlling for age were used to compare the three groups on VCI, PRI, WMI, PSI, and GAI and CPI, respectively. Post-hoc comparisons were carried out using Bonferroni’s method for multiple comparisons. 

With regard to 10 subtest scores, analyses of data distribution revealed non-Gaussian variables. Therefore, non-parametric Mann–Whitney tests were performed to compare the three groups on 10 WAIS-IV subtests. The significance threshold was *p* = 0.05.

## 3. Results

The analysis of the scores at FSIQ showed significant differences among the three groups [*F*(2,91) = 9.08, *p* < 0.001], with the highest FSIQ in the TD group and the lowest FSIQ in the Mixed-SLD group. Post doc analysis revealed significant discrepancies between the TD group (mean score = 113.00, SD = 8.25, range = 98–129) and the Mixed-SLD group (mean score = 100.34, SD = 8.86, range = 83–123; *p* value < 0.001). No significant differences emerged between the TD group and DD group (mean score = 106.25, SD = 10.08, range = 92–122; *p* value = 0.109) and between the DD group and Mixed-SLD group (*p* value = 0.229). All participants showed an FSIQ in the normal range, defined as a standardized score falling within 2 SD below the mean (i.e., <70) [21].

**Figure 1:**
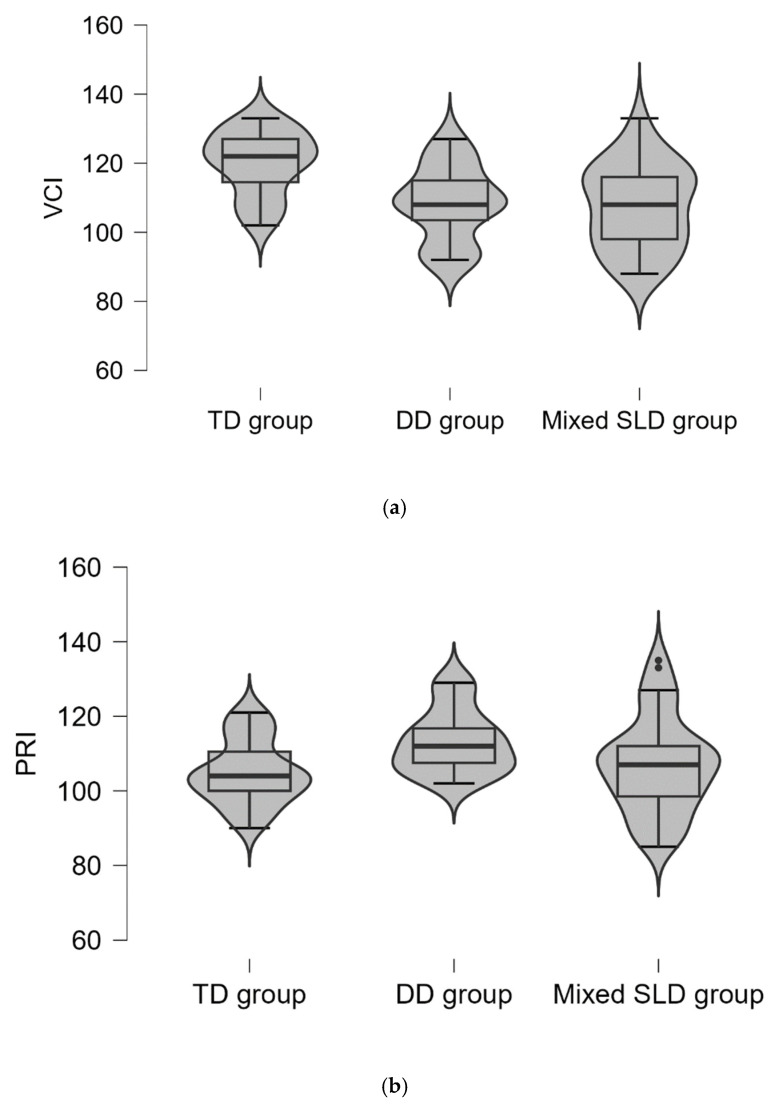

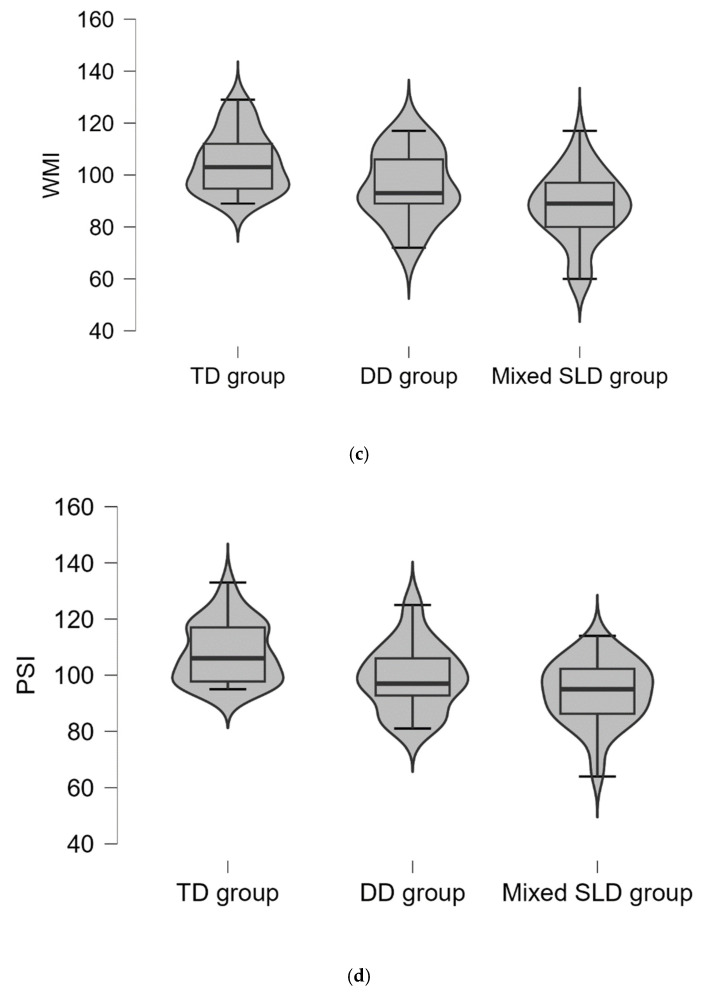
WAIS-IV mean index scores of the TD group (*n* = 38), DD group (*n* = 16), and Mixed-SLD group (*n* = 38). VCI = Verbal Comprehension Index; PRI = Perceptual Reasoning Index; WMI = Working Memory Index; PSI = Processing Speed Index. *(***a***)* shows the Verbal Comprehension Index (VCI); (**b**) shows Perceptual Reasoning Index (PRI); (**c**) shows Working Memory Index (WMI); (**d**) shows Processing Speed Index (PSI).

The descriptive data concerning the VCI, PRI, WMI, and PSI are given in Table 1 and Figure 1. Significant discrepancies emerged among the three groups on all four indexes [VCI: *F*(2,91) = 7.96, *p* = 0.001; PRI: *F*(2,91) = 3.52, *p* = 0.034; WMI: *F*(2,91) = 8.31, *p* < 0.001; PSI: *F*(2,91) = 8.66, *p* < 0.001]. Specifically, VCI and PSI were significantly higher for the TD group than for both the DD group and Mixed-SLD group (see Table 1); the students with Mixed-SLD showed the lowest scores (Table 1). The DD group and the Mixed-SLD group did not significantly differ in VCI and PSI (Table 1). Regarding the PRI, the two groups of students with learning disorder demonstrated higher scores than the TD group; the DD group showed the highest score, with significant differences with respect to TD students (see Table 1). No significant differences emerged between the Mixed-SLD group and both the DD group and the TD group on the PRI (Table 1). With regard to the WMI, the DD and Mixed-type SLD groups demonstrated lower scores than the TD group; the mean score differences were significant only between the Mixed-type SLD group and TD group (see Table 1). No significant differences emerged between the DD group and Mixed-SLD group or between the DD group and TD group (see Table 1).

Examining the percentage of students who fell below the normal range (i.e., <70) across the four indexes, the following data emerged: 10.53% of the students with Mixed-type SLD showed a WMI < 70, and 5.26% of them demonstrated a PSI < 70; there were no deficits in VCI and PSI in this clinical group. In the other two groups, none of the students showed scores below the normal range on the four indexes.

With regard to the GAI and CPI, significant discrepancies emerged among the three groups only on the CPI [GAI: *F*(2,91) = 1.78, *p* = 0.175; CPI: *F*(2,91) = 15.93, *p* < 0.001]. Specifically, the three groups did not differ on the GAI. Both the DD group and the Mixed-SLD group demonstrated significantly lower scores on the CPI relative to the TD group (see Table 2), with the lowest scores in the Mixed-SLD group (Table 2). No significant differences were observed between the DD group and Mixed-SLD group (Table 2).

In the Mixed-type SLD group, 2.63% of students showed scores < 70 on the CPI. 

**Figure 2:**
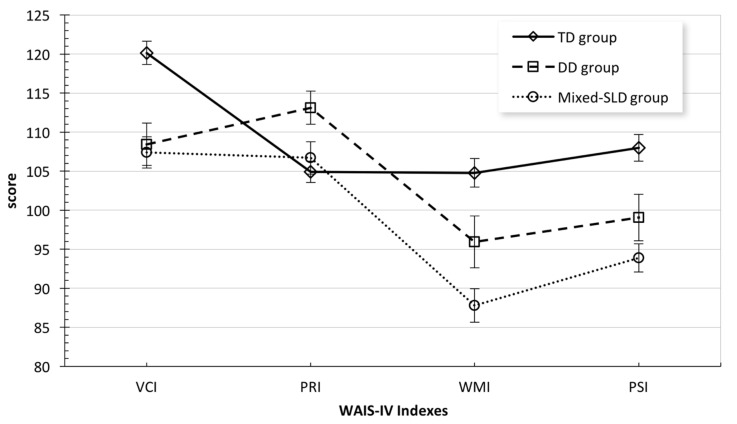
Comparison of the cognitive profiles obtained by the three groups of students in the four WAIS-IV Indexes (standardized mean scores). VCI = Verbal Comprehension Index; PRI = Perceptual Reasoning Index; WMI = Working Memory Index; PSI = Processing Speed Index. Bars indicate the standard errors.

Examining within-group profiles, significant discrepancies among mean WAIS-IV index scores emerged. Specifically, relevant discrepancies were observed in both the DD group and the Mixed-SLD group, between the PRI and WMI and, as result, between GAI and CPI: in both clinical groups the two indexes differed from each other by more than 1 SD (see Figure 2). PRI and PSI also differed from each other by almost 1 SD in both clinical groups (Figure 2). By contrast, the profile of the TD students was more homogenous (Figure 2).

Table 3 shows the WAIS-IV subtests mean scores for each group. Comparing the DD group with the TD group, the VC, SI, DS, CD, and SS subtests mean scores were significantly lower for the DD group than for the TD group (see Table 3); the BD subtest mean score was significantly higher for the DD group than for the TD group (Table 3). Comparing the Mixed-type SLD group with the TD group, the VC, SI, IN, MR, DS, AR, CD, and SS subtests mean scores were significantly lower for the Mixed-type SLD group than for the TD group (see Table 3); the VP subtest mean score was significantly higher for the Mixed-type SLD group than for the TD group (Table 3). Significant differences in the BD, MR, and AR subtests mean scores emerged between the two clinical groups, with higher performances in the DD group (see Table 3).

## 4. Discussion

The purpose of this study was to investigate the cognitive profile of university students with DD and with Mixed-type SLD in order to test hypotheses about cognitive profile features.

Our results indicated significantly lower performances on WAIS-IV VCI, WMI, and PSI in the Mixed-type SLD group, and on VCI and PSI in the DD group, compared to typical students. These results partially contrast with the previous study by Gontkovsky and Golden [52] that, when investigating the cognitive profile in adults with DD between 20 and 62 years of age, found in these individuals significantly lower scores only on the WAIS-IV WMI when compared to the scale’s normative sample. Thus, our findings suggest that different cognitive profiles may emerge in university students with different types of SLD and highlight the importance of studying the university students with SLD specifically.

Interestingly, both the DD group and the Mixed-type SLD group showed a higher PRI than the control group; these differences on the PRI appeared statistically significant only between the DD and TD groups, suggesting high performance in perceptual reasoning tasks in university students with DD. This result appears in line with several studies that investigated the cognitive profile in children [42,44,61] and adults with DD [55,57] and found that dyslexics are characterized by higher visuo-spatial abilities relative to TD peers. This finding seems to also support the idea that, when considering measures of intelligence that include only the core aspects of reasoning, students with SLD may demonstrate higher scores than TD peers [46]. Furthermore, these data underscore the importance of examining SLD-subtypes separately when investigating cognitive features, to better understand differences and similarities among them; this methodological choice represents a strength of the present work, compared to many studies that investigated heterogeneous groups of children or adults with SLD.

Going into more detail and examining the mean scores of the VCI and PRI subtests, TD students significantly outperformed both the DD group on Vocabulary and Similarities, and the Mixed-type SLD group on Vocabulary, Similarities, and Information. Students with DD significantly outperformed the Mixed-type SLD and TD groups on Block Design (+1 SD), and the Mixed-type SLD group on Matrix Reasoning. The Mixed-type SLD group significantly outperformed the TD group on Visual Puzzles but performed significantly worse on Matrix Reasoning. However, although some scores appear significantly lower on some VCI and PRI subtests, especially in the Mixed-type SLD group, the performances on all these subtests are within the average range and therefore cannot be considered impaired, which is in line with the patterns of scores previously reported by Gontkovsky and Golden [52] in their study of adults with learning disorders. It is also interesting to note that both the students with DD and with Mixed-type SLD did not show relevant discrepancies between VCI and PRI; this appears in line with the few studies that, focusing on adults with SLD, found good performance on VCI in those who had a higher education level [55,56,57]. These results suggest that in university students, perceptual reasoning may represent a strength and verbal abilities not necessarily a weakness.

Importantly, lower scores on WMI and PSI with respect to VCI and PRI were observed in both the DD and Mixed-type SLD group. Consistent with previous studies that found similar results in both children [38,39,42,44,61,62] and adults with SLD [52,53,54,57], our study provides new evidence of this discrepancy in university students with DD and Mixed-type SLD. With regard to WMI and PSI, the worse performances were detected in the university students with Mixed-type SLD, with some of these students showing impaired performances, i.e., below 70, in the two indexes (10.53% of them on WMI; 5.26% of them on PSI). No students with DD exhibited these types of deficits and students with DD differed from the TD students only on the PSI. Thus, according with the model of multiple cognitive deficits at the basis of the SLD [7,10,63], our study seems to confirm memory difficulties and slow processing in university students with Mixed-type SLD in particular.

To understand more precisely cognitive difficulties encountered in the two clinical groups, we examined the mean scores of the two subtests of WMI and of PSI. With regard to the WMI subtests, the Digit Span mean scores of the two clinical groups appeared significantly lower than that of the TD group; moreover, the scores obtained on the Digit Span subtest by the two clinical groups were the lowest among all the WAIS-IV subtests with relevant discrepancies relative to other subtests in both groups. Compared to the DD and TD groups, the Mixed-type SLD group significantly underperformed on the Arithmetic Reasoning subtest as well; this result highlights the major processing difficulties that characterize students with multiple learning disorders. Regarding the PSI subtests, both the DD group and Mixed-type SLD group were significantly outperformed by the TD group in both Coding and Symbol Search. These findings are in line with most research that focused on working memory and processing speed in children and adults with learning disabilities [7,9,56,62,64] and suggest that these WAIS-IV subtests are sensitive to processing difficulties in university students with DD and Mixed-type SLD.

These processing difficulties explain the significant differences that we found between the two clinical groups and the TD group on CPI and the relevant discrepancy found between GAI and CPI in both the DD and Mixed-type SLD group. The GAI–CPI discrepancy was more evident in the students with Mixed-type SLD, who showed a CPI 0.73 SD below the normative data. These results are in line with previous research that found a GAI–CPI discrepancy across all SLD subgroups in both children and adults with SLD, but that it was more important in individuals with mixed disorders [38,42,44]. For example, studying the cognitive profile in Italian children with SLD using the WISC-IV, Toffalini and colleagues [44] found that the CPI was only a few points below the normative data in children with reading and spelling disorders, but around 1 SD below in those with mixed disorders. Our findings seem thus to confirm this profile even in university students.

As a result of these processing skills, the Mixed-type SLD group showed a significantly lower FSIQ than controls. In the light of what has been described above, it is evident that this reduction in the FSIQ is most likely due to a decrement in working memory and processing speed abilities. In fact, we found a pattern of FSIQ < GAI performance in the Mixed-type SLD group. According to what has been stated by other authors for the developmental age [38,41,42,47], and which also appears to be applicable for university students with SLD based on our findings, the interpretation of the FSIQ as a measure of global intelligence in clinical settings must be regarded with caution because it may be negatively affected by a decrease in the WMI and the PSI. Therefore, the GAI may be considered as a more appropriate measure of intelligence in these patients relative to the FSIQ [28]. In fact, it must be noted that, in this study, university students with a Mixed-type SLD (like students with DD) did not obtain a lower score on the GAI relative to controls; the mean GAI score of these students (i.e., 107.92) fell above the average mean value of the normative sample (i.e., 100) and it was higher than their own mean FSIQ (i.e., 100.34).

The results of the study confirm the main hypotheses, finding strengths in perceptual reasoning and good verbal comprehension abilities in both the DD and Mixed-type SLD groups, whereas weaknesses in working memory and processing speed indexes lead to a pattern of a better GAI than CPI in both clinical groups. Thus, discrepancies between GAI and CPI, well documented in children with SLD, still manifest in adulthood in university students. Our findings also reveal worse cognitive performance in university students with a mixed learning disorder, relative to students with only a reading deficit.

Although the present study provides new relevant insights, some limitations should be acknowledged. First, the sample size was small; therefore, the generalisability of our findings should be carefully considered. The specific focus on university students with DD and Mixed-type SLD represents an added value to our work; however, further studies need to be conducted with larger samples to generalise our results. Second, we specifically examined the 10 WAIS-IV core subtests, with other important WAIS-IV dimensions not considered, such as Letter-number sequencing, Cancelation, Figure Weights, Comprehension, and Picture Completion. These subtests might provide additional information regarding the cognitive profile of these students. Future research should also further investigate the relationships between these cognitive profiles and other cognitive, relational, and emotional features in this clinical population.

## 5. Conclusions

The present study was the first to examine the cognitive profile in university students with DD and Mixed-type SLD, and offered relevant insights on the generalizability of current descriptions of cognitive profiles derived by the use of intelligence tests. Our data revealed a different cognitive profile in university students with DD and Mixed-type SLD compared with that of healthy students. Both clinical groups demonstrated high perceptual reasoning skills but weaknesses in higher cognitive processes such as working memory and processing speed, with more marked difficulties in the students with Mixed-type SLD. The cognitive features and distinctive subtest profiles that emerged cannot be considered clinically meaningless, and should guide the assessment and the definitions of intervention programs, special educational needs, and strategies of compensation [42]. As for the assessment, our results seem to indicate that the particular discrepancies within the cognitive profile (more than global scores) should be taken into account, in order to be aware of the specific strengths and weaknesses (that vary across SLD subtypes) and offer the best possible support [44]. Our findings also underscore the importance of carrying out individualized and personalized educational interventions for these students, as well as using compensatory tools and applying dispensatory measures. The Italian guidelines for the intervention in children and adults with SLD [58], drawn up on the basis of the most recent scientific knowledge, specify what educational institutions and universities should offer the students with SLD to support their learning and thus to guarantee the right to study. Furthermore, it is known from the literature [17,18,19] that university students with SLD show great psychological suffering related to their leaning difficulties that may affect self-esteem and motivation and may be accompanied by emotional and social difficulties. Recently, a study conducted on Italian students with SLD found a mediating role of cognitive characteristics on the relationship between emotional–behavioral profile and learning impairment [65]. According to D’Elia and colleagues [54], taking into account the specific cognitive profiles of these students also means ensuring a greater understanding of the resources to be enhanced to support not only their cognitive growth, but also their emotional and social well-being.

## Figures and Tables

**Table 1:** Descriptive analyses (standardized mean score, SD, range) and group differences (*p* value of MANOVA controlling for age) on WAIS-IV Indexes scores (VCI, PRI, WMI, and PSI) for the three groups. Significant results are in bold. (1) = Students with Developmental Dyslexia (DD); (2) = Students with Mixed-SLD; (3) = Tipically Developing (TD) Students.

	Students with DD (1) (*n* = 16)		Students with Mixed-SLD (2) (*n* = 38)		TD Students (3) (*n* = 38)		MANOVA		
*WAIS-IV Indexes*	Mean (SD)	Range	Mean (SD)	Range	Mean (SD)	Range	p (1–2 *)	p (1–3 *)	p (2–3 *)
Verbal Comprehension Index (VCI)	108.44 (10.86)	92–127	107.42 (12.25)	88–133	120.16 (9.14)	102–133	1.00	**0.005**	**0.002**
Perceptual Reasoning Index (PRI)	113.13 (8.47)	102–129	106.71 (12.71)	85–135	104.92 (8.53)	90–121	0.195	**0.031**	1.00
Working Memory Index (WMI)	95.94 (13.19)	72–117	87.79 (13.29)	60–117	104.79 (11.26)	89–129	0.239	0.153	**<0.001**
Processing Speed Index (PSI)	90.06 (11.94)	81–125	93.89 (11.15)	64–114	108.00 (10.57)	95–133	0.552	**0.049**	**<0.001**

* The numbers in brackets mean the comparison groups.

**Table 2:** Descriptive analyses (standardized mean score, SD, range) and group differences (*p* value of MANOVA controlling for age) on WAIS-IV additional indexes scores (GAI and CPI) for the three groups. Significant results are in bold.

	Students with DD (1) (*n* = 16)		Students with Mixed-SLD (2) (*n* = 38)		TD Students (3) (*n* = 38)		MANOVA		
*WAIS-IV Additional Indexes*	Mean (SD)	Range	Mean (SD)	Range	Mean (SD)	Range	p (1–2 *)	p (1–3 *)	p (2–3 *)
General Ability Index (GAI)	111.31 (9.49)	93–128	107.92 (10.14)	90–133	114.50 (8.06)	98–130	1.00	1.00	0.188
Cognitive Proficiency Index (CPI)	96.94 (11.76)	78–118	89.00 (10.14)	70–112	107.39 (9.40)	92–128	0.097	**0.010**	**<0.001**

* The numbers in brackets mean the comparison groups.

**Table 3:** Descriptive analyses (standardized mean score, SD, range) and group differences (Mann–Whitney test) on WAIS-IV subtest scores for the three groups. Significant results are in bold.

	Students with DD (1) (*n* = 16)		Students with Mixed-SLD (2) (*n* = 38)		TD Students (3) (*n* = 38)		Mann–Whitney Test		
*WAIS-IV Subtests*	Mean (SD)	Range	Mean (SD)	Range	Mean (SD)	Range	U/p (1–2*)	U/p (1–3 *)	U/p (2–3 *)
Verbal Comprehension Index (VCI)									
Vocabulary (VC)	11.31 (2.68)	7–17	11.42 (2.31)	7–17	13.61 (2.21)	10–19	91/0.804	155/**0.004**	362/**<0.001**
Information (IN)	10.19 (2.83)	6–15	10.24 (2.53)	5–15	11.89 (2.54)	6–17	302.5/0.977	208/0.067	489/**0.015**
Similarities (SI)	12.63 (1.89)	11–17	12.13 (2.98)	7–19	14.89 (1.97)	10–19	257/0.367	120.5/**<0.001**	312.5/**<0.001**
Perceptual Reasoning Index (PRI)									
Block Design (BD)	13.00 (2.16)	9–17	10.84 (2.59)	6–18	10.24 (2.62)	5–15	154.5/**0.004**	131/**0.001**	653.5/0.473
Matrix Reasoning (MR)	12.00 (2.28)	8–16	10.37 (2.27)	6–15	11.50 (1.81)	8–15	175.5/**0.014**	246.5/0.266	466.5/**0.007**
Visual Puzzles (VP)	11.50 (1.97)	8–15	11.71 (2.91)	5–17	10.66 (1.94)	7–14	278/0.619	234.5/0.183	529/**0.043**
Working Memory Index (WMI)									
Digit Span (DS)	8.44 (3.16)	3–14	7.63 (3.10)	3–14	10.68 (2.18)	7–15	260.5/0.407	171.5/**0.011**	328/**<0.001**
Arithmetic Reasoning (AR)	10.13 (2.53)	7–16	8.00 (2.57)	3–13	10.95 (2.37)	7–17	168/**0.009**	231/0.154	275/**<0.001**
Processing Speed Index (PSI)									
Coding (CD)	9.81 (2.07)	7–14	9.08 (2.21)	4–14	11.37 (2.08)	8–15	250/0.302	184.5/**0.022**	342/**<0.001**
Symbol Search (SS)	9.81 (2.79)	5–17	8.71 (2.29)	3–13	11.47 (2.41)	8–17	240.5/0.224	194.5/**0.036**	304/**<0.001**

* The numbers in brackets mean the comparison groups.

## Data Availability

The datasets generated for this study are available on request to the corresponding author.
